# When public action undermines public health: a critical examination of antifluoridationist literature

**DOI:** 10.1186/1743-8462-4-25

**Published:** 2007-12-09

**Authors:** Jason M Armfield

**Affiliations:** 1 Australian Research Centre for Population Oral Health, School of Dentistry, University of Adelaide, South Australia, Australia

## Abstract

**Background:**

The addition of the chemical fluorine to the water supply, called water fluoridation, reduces dental caries by making teeth more resistant to demineralisation and more likely to remineralise when initially decayed. This process has been implemented in more than 30 countries around the world, is cost-effective and has been shown to be efficacious in preventing decay across a person's lifespan. However, attempts to expand this major public health achievement in line with Australia's National Oral Health Plan 2004–2013 are almost universally met with considerable resistance from opponents of water fluoridation, who engage in coordinated campaigns to portray water fluoridation as ineffective and highly dangerous.

**Discussion:**

Water fluoridation opponents employ multiple techniques to try and undermine the scientifically established effectiveness of water fluoridation. The materials they use are often based on Internet resources or published books that present a highly misleading picture of water fluoridation. These materials are used to sway public and political opinion to the detriment of public health. Despite an extensive body of literature, both studies and results within studies are often selectively reported, giving a biased portrayal of water fluoridation effectiveness. Positive findings are downplayed or trivialised and the population implications of these findings misinterpreted. Ecological comparisons are sometimes used to support spurious conclusions. Opponents of water fluoridation frequently repeat that water fluoridation is associated with adverse health effects and studies are selectively picked from the extensive literature to convey only claimed adverse findings related to water fluoridation. Techniques such as "the big lie" and innuendo are used to associate water fluoridation with health and environmental disasters, without factual support. Half-truths are presented, fallacious statements reiterated, and attempts are made to bamboozle the public with a large list of claims and quotes often with little scientific basis. Ultimately, attempts are made to discredit and slander scientists and various health organisations that support water fluoridation.

**Summary:**

Water fluoridation is an important public health initiative that has been found to be safe and effective. Nonetheless, the implementation of water fluoridation is still regularly interrupted by a relatively small group of individuals who use misinformation and rhetoric to induce doubts in the minds of the public and government officials. It is important that public health officials are aware of these tactics so that they can better counter their negative effect.

## Background

The addition of fluorine to the water supply, termed water fluoridation, is carried out as a public health measure to improve oral health. One of the ways fluorine confers its benefit is by changing the crystalline structure of teeth. Fluorine ions replace hydroxide ions in calcium hydroxyapatite, Ca_5_{(PO_4_)_3_OH}, in teeth, forming calcium fluoroapatite, Ca_5_{(PO_4_)_3_F}, which is more chemically stable and more resistant to acid attack than calcium hydroxyapatite [1]. However, as well as making the enamel more resistant to acid attack by altering the chemical structure of the enamel, fluoride helps to protect teeth by promoting the remineralisation of early decayed lesions and by reducing the ability of the bacteria on the teeth to produce acid.

Water fluoridation was first carried out in the USA after studies by Dean [2,3] found that higher levels of fluoride (fluorine when part of a chemical compound) in the water supply appeared to confer a caries preventive effect. Since then, water fluoridation has been adopted in over 30 countries, reaching an estimated 350 million people worldwide [4]. Indeed, the fluoridation of drinking water to control dental disease has been referred to by the US Centers for Disease Control as one of the Top 10 public health achievements of the 20th century [5]. Fluoridated water reaches people from all socio-economic strata of society [6-8] helping to erode the socio-economic gradient in oral disease experience. It is efficient, cost-effective and considered the single most effective means of preventing tooth decay over a person's lifetime [9]. In addition, water fluoridation alters the dynamics of decay initiation. Rather than just affecting an individual at a point in time, water fluoridation reduces the incidence of decay. This is a phenomenon played out over a person's lifetime. The effect of water fluoridation is not a static 'one-off' benefit.

Oral health is fundamental to overall health, yet many adult Australians and a significant percentage of children still suffer persistent high levels of oral disease and disability. As a response to a report released in 2001 on the 'Oral Health of Australians' [10], the National Advisory Committee on Oral Health, established by the Australian Health Minister's Conference, signed off on 'Australia's National Oral Health Plan 2004–2013' [11]. One of the four broad themes underpinning the plan was the need to adopt a population health approach, with a strong focus on oral health promotion and the early identification and treatment of oral disease. Although most dental services are provided within the private sector, both the Commonwealth and the States and Territory governments can and do provide financial support for dental services to certain sections of the population. State and Territory governments are also responsible for the implementation of water fluoridation under a raft of different legislative arrangements, although in practice, and for various reasons, those responsibilities are often devolved to local councils. One of the short term goals of population oral health promotion under the National Oral Health Plan was the extension of the fluoridation of public water supplies to all communities across Australia with populations of 1000 people or more. The suggested population cut-off was based on research from New Zealand which showed that this was the practical lower bound at which water fluoridation remained cost-effective [12].

## Discussion

Partially in response to the objective of extending water fluoridation announced in the National Oral Health Plan, there has been renewed advocacy at the State and Territory level for fluoride to be added to public waters to improve oral health. Nonetheless, this major public health initiative continues to meet considerable opposition where ever it is mooted. Such a response is not confined to Australia. Attempts worldwide to introduce water fluoridation are often thwarted [13,14]. In the US and Canada, for example, one antifluoridation organisation claims that more than 150 communities have rejected water fluoridation in referenda since 1990 [15] while between 1989 and 1994 just over 40% of referenda were defeated in the US [13].

Efforts to introduce water fluoridation are almost universally met with a coordinated campaign involving newspaper articles, calls to talkback radio, letterbox leaflet drops, public forums and community agitation. Proponents of water fluoridation are invited to public forums to debate against fluoridation opponents and these are often given prominent media coverage. If advocates of water fluoridation attend these debates they may be bombarded with one claim after another of which they have no hope of adequately addressing in the limited opportunities afforded in a public debating match [16]. If, however, water fluoridation proponents decline to attend public fluoride debates they are labelled as being arrogant, condescending and contemptuous [17] and their desire to not attend lambasted as "an insult to both science and democracy" [18]. Opponents of water fluoridation make the inaccurate claim that public debates are a forum for "open, rational, scientific argument and evidence" and that by refusing to attend debates proponents of water fluoridation are maintaining the process by using political power and influence [19].

Statements regarding the scientific controversy surrounding water fluoridation are generally regarded as artefacts of antifluoridationist activity, with actual scientific debate over water fluoridation being resolved decades ago. Almost all major dental and health organisations either support water fluoridation or have found no association between it and adverse health effects [20]. Nonetheless, propagating the idea of an ongoing scientific debate gives the illusion of scientific uncertainty and is a favoured tactic of water fluoridation opponents. In 1978, *Consumer Reports *published a two-part series on fluoridation that concluded:

The simple truth is that there's no "scientific controversy" over the safety of fluoridation. The practice is safe, economical, and beneficial. The survival of this fake controversy represents, in Consumers Union's opinion, one of the major triumphs of quackery over science in our generation." [21]

And yet, more than a quarter of a century after these words were printed the manufactured 'controversy' shows no signs of diminishing.

In the US, those opposed to water fluoridation have been described as a heterogenous group and range from well-intentioned and concerned citizens to professional activists to extremists [22]. In the 1980s, Hastreiter argued that the leaders of the movement in the US were "individuals who are marginal to the social, psychological, political, and professional mainstream" [23]. Also involved in water fluoridation opposition in most countries are companies selling bottled water and water filters, purveyors of alternative medicines and therapies, and some environmental scientists. Opponents of water fluoridation share the characteristic of being highly mobilised and organised and rely heavily on propagating their opinion via the popular media, which is often willing, if not keen, to publish their sensationalist claims. While provocative and emotive arguments are commonly aired in the media, the ability of water fluoridation opponents to delay or halt the introduction of water fluoridation though their public lobbying campaigns represents a serious and detrimental public health outcome. Campaigns are often based on information available on anti-fluoride websites and are often spearheaded by one of a small number of ardent fluoridation opponents.

Scientific journals provide an essential role in both information sharing and as a forum for scientific debate. The process of peer review in these journals helps ensure that poor quality research is rejected, unsupported conclusions are censured, and ascientific speculation is appropriately identified. With the advent of the World Wide Web, however, opinion can be propagated from any web site, by anyone, to reach a potential audience of millions.

Because Internet resources are increasingly being used by the public as a source for dental and general health information [24-26], the uncontrolled spread of information on the Internet has led to concern over its appropriateness and quality. Water fluoridation information on the World Wide Web is presented to the public indiscriminately and has been found to range from factual, to unsubstantiated opinion, to outright fraud [27]. Although the overwhelming majority of scientific enquiry supports the benefits of water fluoridation, members of the public who type the term "water fluoridation" into any of the major search engines would immediately be presented with a disproportionate percentage of anti-fluoridation websites (Table 1). If a concerned member of the public were to type in the search term "water fluoridation dangers" almost the only information presented to them would reflect an antifluoride perspective.

**Table 1:** Classification of first 20 results based on Internet searches for "water fluoridation" and "water fluoridation dangers" using five major search engines

	"water fluoridation"				"water fluoridation dangers"			
		
Search engine	For	Against	Reviews	Other	For	Against	Reviews	Other
Google	7	9	3	1	0	20	0	0
Yahoo	5	11	1	3	0	19	0	1
MSN	8	10	1	1	1	18	0	1
AOL	6	9	3	2	0	20	0	0
Ask	3	16	1	0	0	20	0	0

Note: Search conducted on 10 April 2006, from Australia. Listed search engines account for 90.3% of all US Internet searches (AC Nielson, 2005)

Adding to the one-sided presentation of information on water fluoridation on the Internet is a bias in media reporting. Although the media have a social responsibility to inform the public of possible impending dangers [28], they are often poorly equipped to adequately explain the underlying complexities of risk issues in science [29]. There is a payoff for generating controversy in an increased audience. In 2001, the Dental Health Foundation in Ireland analysed 240 recent print, radio and television articles relating to water fluoridation over a one-year period, finding the media coverage to be predominantly negative (52%) with only 14% of articles judged as being positive [30]. A similar bias was found with press cuttings in the UK [31]. Easley explains this bias as demonstrating subversion of the media; that is, winning over the media by presenting controversy, sensationalist claims and a David vs Goliath concept which appeals to people's proclivity to support the 'underdog' in conflicts [16]. Radio talk shows, an increasingly powerful force in US politics, have been found to present a barrage of negativity about water fluoridation and have been known to screen out supportive viewpoints so that only a one-sided view reaches the public [13].

Researchers in Ireland argue that the high percentage of negative coverage most likely stems from its increased dramatic and sensationalist appeal, providing a payoff in an increased readership or audience [30]. Of course, concerns regarding the content of media coverage effect not just water fluoridation but other public health strategies. For example, an analysis of the print media's coverage of heroin prescription trials [32], compulsory vaccinations [33] and many other public health and health related issues reveals similar bias. Of concern is that consumers may more readily recall this negative media coverage than the positive coverage.

The major public health implications of the spread of misinformation regarding water fluoridation on the Internet is that this information finds its way into local anti-fluoride campaign materials which are used to influence councils who ultimately are required to make decisions on water fluoridation implementation. The standard procedure for making council decisions on matters outside of the expertise of council members is to invite comment from representatives of different sides of the issue. Councillors are presented with conflicting information of which they are not qualified to judge, and under public pressure by a small number of committed activists, may decide to maintain the status quo which means to not introduce water fluoridation. Other councillors may decide to carry out their own research and may turn to the Internet which is the primary source of misinformation regarding the fluoridation of water. One councillor from Northern NSW has quipped that "It took five minutes of research to confirm my opinion about fluoride" [34].

Public plebiscites are also frequently adopted by councillors as a solution to not having to make what is sometimes viewed as an unpopular decision, and anti-fluoride misinformation is used to sway the public opinion in this scenario as well. For example, a leaflet distributed by a rural council area in NSW prior to a public plebiscite on the addition of fluoride to the town water supply claimed that water fluoridation was unethical, unsafe and ineffective [35]. That the antifluoride rhetoric was given the same space as for the 'Yes campaign' puts these viewpoints on a par and gives the impression that both viewpoints have equal weight, despite the fact that no credible public health, dental or medical organisations anywhere in the world are opposed to water fluoridation. Opponents of water fluoridation in Australia make extensive use of the steady stream of ready-made misinformation available from overseas sources.

In modern democracies it is vital that choices be informed, and scientific evidence is critical for this to occur. Scientific evidence forms the fundamental bedrock for decision making for public health practitioners, but the process for much of the population is more complex with any decision based on a range of opinions, beliefs, emotions, risk assessments, and experiences. Indeed, it has been argued that appealing to facts, and to accredited experts for their interpretation, has been increasingly compromised by an awareness of the limitations of experts and expert knowledge in resolving issues of public controversy [36]. Further, it is believed that there is a growing public perception that experts can and do disagree and that purportedly "disinterested" advice may be influenced by economic, professional, or political considerations [36]. Such perceptions are attributed partly for the increased resistance of the general public to health promotion messages and interventions [37]. At the same time, levels of cynicism regarding politicians are at an historic high [38]. Unfortunately, decisions to do with the implementation of water fluoridation are often subverted to political purposes. Politicians as well as opposition groups are quick to pick up on rhetoric which may resonate with the public, with resistance to public health measures often having at their heart an appeal to an individualistic ideology that valourises the fight against the erosion of civil liberties and promotes suspicion of 'faceless' authority figures [39].

In population public health there is frequently a tension between public good and individual freedoms. Kass, for instance, has described the dilemma faced by the population-based focus of public health concerning the infringement on individual liberties in ethically troublesome ways [40,41]. The introduction of bans on public smoking, the requirements to wear seatbelts in cares or helmets on motorcycles, and compulsory childhood immunisations all infringe to some extent on personal choice, yet all are supported by public health advocates and backed by government legislation. The ethics behind this process is now well established, and generally accepted by the community. This perhaps explains why a significant majority of the Australian population continue to support the practice of water fluoridation [42,43].

It should be noted that antagonism towards and opposition to the views of public health practitioners by minority groups is not restricted to the fight over water fluoridation. Parallels can be drawn with some other public health controversies such as compulsory child immunisations or the use of genetically modified (GM) foods. There is now, for example, an extensive body of research analysing the debate over the safety of the MMR (measles, mumps, and rubella) vaccine [44-48]. Concerns over the MMR vaccine first surfaced following a study by Wakefield et al. published in the Lancet linking the vaccine to autism [49]. The study prompted widespread concern and the resultant controversy has been blamed on a significant decrease in vaccination rates in the UK [48,50] and to a lesser extent in Australia and the US [45]. Fitzpatrick has argued that the perceptions of risk, choice and chance are central to the public's response to the controversy [46] and as a result of these concerns a number of groups have formed to oppose the compulsory MMR immunisation of children. However, while a number of anti-vaccination websites do exist [51,52], concerns over vaccinations are believed to have been led more by the media in response to the Wakefield et al. study than by organised or influential opposition groups as with water fluoridation [48,53].

While the moral, ethical and social concerns over water fluoridation are both legitimate and fully deserving of further investigation, they lie outside of the intent of this current paper. Instead, this paper will restrict its analysis to a critique of antifluoridationist literature. Rather than attempt a rebuttal to every claim and research finding put forward by water fluoridation opponents, a task attempted elsewhere [20,54,55], an analysis of the anti-fluoride lobby's techniques and tactics will be pursued. It is hoped that this may achieve two purposes: (1) to aid health and public health officials in countering the antifluoridationist strategies; and (2) to provide information to help both the general public and public health advocates sort the truth from the fiction and learn to identify the use of rhetoric, misinformation and misrepresentation relating to water fluoridation. Indeed, the irony of water fluoridation opponents claim that "fluoridation is an issue where the scientific method and principles are being set aside by public health authorities" [56] is that nowhere is the scientific approach more blatantly flouted than within anti-fluoridation literature.

### Denying the benefits of water fluoridation

One of the fundamental tactics of water fluoridation opponents is to either deny or to besmirch the benefits of water fluoridation. It is argued that water fluoridation is either not effective or, at best, only minimally effective [57,58]. It has even been argued that water fluoridation actually harms teeth, making them more susceptible to caries [59]. These claims have been adequately addressed elsewhere with numerous systematic reviews finding that water fluoridation is associated with improved oral health [60-62]. Nonetheless, opponents of water fluoridation use several techniques to try and mislead the public in terms of the effectiveness of water fluoridation.

#### Selective reporting of studies

Each year hundreds of studies are published in the scientific literature regarding the effects of fluoride on animals and humans. In order to examine a relationship between variables across an extensive body of literature scientists often make use of literature reviews or meta-analyses. Water fluoridation opponents, however, take a contrary approach. Rather than trying to discern a given outcome for fluoride exposure across all available studies, they handpick studies to cite. Findings not supporting their viewpoint are entirely disregarded while other findings may be prominently utilised. As an example, the York report, a large and comprehensive systematic review of the water fluoridation literature published in 2000, found that of the 29 studies included that examined the relationship between incidence of bone fracture and water fluoridation, four indicated a significant increased risk of fracture, five indicated a significant decrease in risk of fracture, while the other studies found no significant associations [60]. An article by Kauffman, however, stated that one of the harmful effects of water fluoridation is bone fracture [63] with this contention based on a single published study from the systematic review [64]. While the cited study does represent one of the four studies identified in the York Report as indicating increased risk, no mention is made of the studies finding lower incidence of fractures with water fluoridation nor is any mention made of the 20 studies that failed to find a significant result in any direction.

#### Selective reporting of results

To make the selective reporting of studies even more misleading, often specific results within specific studies are reported while any disconfirming results are ignored. For example, in a study examining the relationship between children's caries experience and consumption of non-fluoridated bottle and tank water [65] the lack of a statistically significant effect on the permanent tooth decay experience of 12-year-olds was the source of numerous articles, paid for press releases, and world-wide presentations. Water fluoridation opponents cite the study as evidence that water fluoridation is ineffective [66,67]. However, the finding of a strong beneficial effect of water fluoridation on the caries experience of younger children's teeth has been entirely ignored. Interestingly, while Pollick [68] in his article 'Scientific evidence continues to support fluoridation of public water supplies' cites this study as supporting water fluoridation, Connett [69] uses the same study to support a diametrically opposed view in his paper 'Scientific evidence fails to support fluoridation of public water supplies'.

#### Downplaying or ignoring the evidence

Water fluoridation opponents claim that there is either no 'significant' or no 'substantial' reduction in tooth decay resulting from exposure to fluoridated water [57]. Numerous studies are cited showing no difference, many of which simply compare one community with another without any control for other possible variations between those communities. Systematic reviews, such as the York report [60], which include no studies classified by its criteria as Level A, are cited as supposed proof of the total absence of high quality evidence [59], confusing the concept of quality with the York report's evidence classification. Reductions of 'a fraction of one decayed tooth per child' are dismissed as not substantial. Finally, findings of reductions in decay experience in non-fluoridated areas are used as evidence that fluoridation provides no added benefit to changes occurring in the absence of water fluoridation [59,70]. All of these arguments, however, are flawed and misrepresent study results.

Water fluoridation is a population-level caries preventive strategy. Therefore, the appropriate method of measuring effectiveness is to look at the population level effect rather than look at the effect on any given individual. The York report's systematic review [60] found reductions of between 0.5 and 4.4 decayed, missing and filled teeth per child on average. Reductions of between 20% and 40% have elsewhere been commonly reported. Differences of between 32% and 55% in the deciduous teeth and 20% and 65% in the permanent teeth have been reported in Australia [71]. Contrary to claims made by opponents of water fluoridation, these differences have been found to be statistically significant in published scientific research. In addition, although the generally low caries levels in Australia and some other countries might make such percentage differences work out to only a fraction of a tooth per child, at a population level this equates to a tremendous reduction in the amount of disease present. For instance, a recent Australian study found that across socio-economic groups water fluoridation was associated with caries reductions of between 48% and 75% [72]. However, in a paid-for press release by the New York State Coalition Opposed to Fluoridation (NYSCOF), Paul Beeber, President of the NYSCOF, argued that this indicates a waste of taxpayers money "for such a slim, if any, benefit" [73]. While a difference in decay experience between fluoridated and non-fluoridated areas of 0.7 teeth on average might be dismissed by anti-fluoridation lobby groups as "meagre" [73], if this finding could be extended across the Australian child population of 1.8 million children, it would translate into over a million teeth saved from decay, affecting hundreds of thousands of children. Such a result would be significant in terms of the extra disease prevented, the associated reduction in suffering, and savings in treatment costs.

Another example of downplaying the evidence of the effectiveness of water fluoridation is the argument that fluoridated water is not required to be ingested to be effective. Opponents of water fluoridation often present quotes by researchers saying that the primary effect of fluoride is topical (that is acting on the tooth surface) rather than systemic [59]. However, recent research in Australia by Singh and colleagues [74-76] has found that the pre-eruptive or systemic effect of fluoride in water supplies is at least as important in accounting for the caries preventive effect of consumption of fluoridated water as the post-eruptive or topical effect. It is common for opponents of water fluoridation to cling to old or out-of-date research while ignoring newer research that might cast doubt on their theories. Sometimes statistics and results from many decades ago are quoted to support their beliefs and statements.

#### Using ecological comparisons

Another ploy of water fluoridation opponents is to use ecological comparisons in an effort to demonstrate that water fluoridation is ineffective. With this tactic, the decay experience of children in a specific fluoridated area is compared unfavourably to that of children in a specific non-fluoridated area [59,77]. Despite such ecological comparisons providing a poor level of evidence due to their inability to take into account other variations between the areas which are also related to dental health (such as differences in diet, socio-economic status, exposure to discretionary fluorides, and oral health behaviours) this type of 'evidence' has been frequently used to shore up the arguments of water fluoridation opponents [72]. Although selected associations such as these provide no evidence of causality, many people may be inclined to accept ecological comparisons as a valid test of the effectiveness of water fluoridation and opponents of water fluoridation continue to use this approach to mislead the public and government officials.

### Fear mongering

One of the easiest ways to preserve the status quo is by raising potentially dangerous or fearful consequences associated with possible change. This technique is common in politics where allusion to personal impropriety or dire economic consequences may be enough to taint a political candidate or party. Water fluoridation opponents, like politicians, make extensive use of fear mongering. Fluoride exposure has been linked in the antifluoridationist literature to poisonings and various accidents, allergies, brain dysfunctions such as Alzheimer's disease, hyperactivity, low intelligence, arthritis, bone diseases including hip fractures and osteosarcomas, cancers, dental fluorosis, gastrointestinal problems, diseases of the kidney, pineal gland and thyroid gland, reproductive issues, AIDS, and even with increased tooth decay [20]. Links have been made between fluoride consumption and birth defects, perinatal deaths and increased crime. Claims that governments are using water fluoridation to 'dumb down' the population [78-80], help the spread of communism [81], or prepare the way for the New World Order [81,82] are used occasionally in antifluoridationist writings. A consistent thread is that those scientists and government officials who are pro-fluoride are under the sway of multi-national corporations or funded to support water fluoridation.

Despite the extensive claims of water fluoridation opponents, the only substantiated link between fluoride exposure and any health side effect is for dental fluorosis [60,61]. This condition involves a hypomineralisation of the tooth surface in contrast to the demineralisation of the tooth surface associated with decay. In addition, there is no evidence that there is any financial remuneration from the sugar or aluminium industry for scientists publishing materials that show the benefits of water fluoridation.

#### Misrepresentation of the truth

In many cases information is misrepresented in order to support the anti-fluoride argument. Misrepresentation involves taking information out of the context in which it is presented in order to make it support a viewpoint which the author or authors did not intend. Statements are taken out of context, and results are selectively reported. In Australia, for example, opponents of water fluoridation make the false claim that the National Health and Medical Research Council (NHMRC) recommends "that NO additional fluoride be given to children under three years" [59]. It is argued that there is a contradiction between this claimed recommendation and support by the NHMRC for water fluoridation. However, the NHMRC actually recommends that no fluoride supplements be given to children under three years of age [62]. Fluoride supplements are tablets or drops, often available from a chemist, used to increase intake of fluoride in non-fluoridated areas. There is, therefore, no contradiction with the NHMRC supporting water fluoridation. Yet, such misrepresentations continue to be made.

Misrepresentation often takes place by omission. Connett [83,84], for example, has regularly cited a study from China [85] as finding a doubling of hip fractures when people consume water with 1.5 ppm fluoride and a tripling of fractures when consuming water of greater than 4.3 ppm fluoride. This is cited as evidence of the deleterious effect of water fluoridation on the bones. What Connett does not state is that the doubling of hip fractures at 1.5ppm is not statistically significant and that the authors' find a 'U' shaped relationship between the amount of fluoride in the water and fractures, with optimally fluoridated water actually conferring a protective effect on bone fractures. Yet, handpicked and misrepresentative information may find its way from the Internet to prominent pieces in national newspapers [86] with little regard for the truth.

#### The big lie

Bernhardt and Sprague have argued that the basic technique of antifluoridationists is to make the claim that water fluoridation causes a number of serious ailments that people fear [87]. This technique involves telling a lie so large that it defies anyone to believe that someone would distort the truth to such an extreme extent, and is aided in its effectiveness by constant repetition. Research findings indicate that if something is said often enough people will tend to think there is some truth in it, a process now called the illusory-truth effect [88]. Further to this technique is what is called the 'laundry list' approach, listing so many 'evils' that even if water fluoridation proponents can adequately respond to some they can not address all [89]. Such a technique is particularly effective in debates, letters to the editor or in the popular media where the time and opportunity to reply is limited or non-existent. The American Dental Association catalogues about 30 adverse health effects linked in anti-fluoridation literature to water fluoridation [20].

#### Half-truths

A half-truth is a statement that is only partly true and is generally intended to deceive. If an uninformed member of the public were to read and believe the following text, taken from an anti-fluoride website, they might have good grounds for being concerned about water fluoridation:

"Did you know that sodium Fluoride is ... one of the basic ingredients in both PROZAC (Fluoxetene Hydrochloride) and Sarin Nerve Gas (Isopropyl-Methyl-Phosphoryl FLUORIDE) – (Yes, folks the same Sarin Nerve Gas that terrorists released on a crowded Japanese subway train!). Let me repeat: the truth the American public needs to understand is the fact that Sodium Fluoride is nothing more (or less) than a hazardous waste by-product of the nuclear and aluminium industries. In addition to being the primary ingredient in rat and cockroach poisons, it is also a main ingredient in anesthetic, hypnotic, and psychiatric drugs as well as military NERVE GAS! Why, oh why then is it allowed to be added to the toothpastes and drinking water of the American people?" [78]

People may not normally consider that many substances can be harmful or poisonous depending upon the dose. Excessive intake of vitamin D, salt or even water may result in poisoning. The issue of dosage and its relationship to toxicity is rarely mentioned in antifluoridationist rhetoric because it undermines the intended link between water fluoridation and harm. The inclusion of a substance in a poison or toxin does not mean that at smaller doses in humans the substance is still toxic. For example, Warfarin, an anti-coagulant which is the active ingredient in the common rat poison, RatSAK, is also used as a medicine for people in danger of stroke [90,91] and in cases of deep-vein thrombosis [92,93]. The toxic Sarin gas (C_4_H_10_FO_2_P), contains not just fluoride but oxygen, hydrogen and carbon. The fact that a given substance is toxic does not mean that every element contributing to it is also toxic.

#### Innuendo

Innuendo involves an indirect or subtle, usually derogatory, implication in expression. Water fluoridation opponents often link water fluoridation to other medical and government sanctioned practices that have led to aversive and unexpected consequences. An example is thalidomide, a drug that was prescribed to pregnant women during the late 1950s and 1960s to aid sleeping, morning sickness and other pregnancy symptoms and was later found to be teratogenic in foetal development, causing physical deformities [94,95]. Statements such as "When the truth about fluoridation is finally exposed, it may well dwarf the thalidomide tragedy", attributed to Albert Schatz and published by the New Zealand Fluoride Action Network [96], is an example of the use of innuendo. Similarly, Bryson writes "It was an era of thalidomide and plutonium; school segregation and human experimentation; ... atmospheric Hbomb testing and DDT...Fluoridated water was idealized as the ultimate form of 1950's failsafe social engineering" [97]. Again, water fluoridation is linked to a number of dangerous and now controversial practices in an attempt to discredit it by association. The question: "Can we not learn from past assurances of the safety of DDT, thalidomide, and the hundreds of other 'safe' chemicals?" [98], uses innuendo to imply that water fluoridation is also an environmental disaster waiting to happen. It should be realised that this rhetorical practice is intended to sway the opinion of an audience and presents no evidence indicating that water fluoridation causes harm. Thousands of drugs, medicines and chemicals have proven safe and effective and have led to a longer and better quality life. Only a small number of medical substances have proven harmful in the long run and there is no validity in using these as evidence for the danger of any other substance.

#### Follow the leader

Opponents of water fluoridation, despite arguing that water fluoridation should not be introduced just because other areas have implemented it, argue that it should be rejected in Australia in the same way that it has been allegedly rejected by 98% of Western Europe [99]. They state that Austria, Belgium, Denmark, Finland, France, Germany, Italy, the Netherlands, Norway, Sweden and large sectors of the United Kingdom do not have water fluoridation in place and directly or by insinuation make the argument that there must be something wrong with water fluoridation for these countries to have not implemented the practice. This argument is flawed for two reasons. First, it is equally applicable to argue that because the United States, Australia, New Zealand, Ireland, Brazil, some sectors of the United Kingdom, and various other countries have introduced water fluoridation then other nonfluoridated countries should alsointroduce the process. The second is that countries that do not have water fluoridation have mostly not rejected the benefits or science of water fluoridation but have not introduced water fluoridation for a range of other reasons [4]. These have to do with cost, the use of other population preventive practices such as salt fluoridation or the belief that water fluoridation is unnecessary because universal and extensive dental care programs are already in place.

#### Enforced medication

Antifluoridationist literature is replete with scare words, such as "pollutant", "chemical" and "toxic waste" that reinforce the idea of harm. The idea of "enforced medication" is another expression that comes up repeatedly when efforts are made to extend water fluoridation to non-fluoridated areas. According to this argument, fluoride is a medicine, taking medicine should only be a function of individual choice, and therefore water fluoridation is an impingement on our freedom of choice. Use of the term medicine implies something which should only be administered by a doctor acting for the good of an individual. Terminology such as "mass medication" or "forced medication" is often picked up on and used by local government officials who are responsible for decision making [100,101].

The rejoinder to this line of argument is that fluoride added to the water supply is not a medicine. In Australia, for example, fluoride only appears in the Standard for the Uniform Scheduling of Drugs and Poisons as a schedulable substance when used in amounts of more than 1000ppm. In any event, such a population preventive strategy is certainly not without precedent. Iodine is added to salt to help prevent goiter and low intelligence. Folate is added to bread and rice products because of its importance in the development of babies. Vitamins, minerals and other additives are now commonplace in many foods because they are believed to confer health benefits. While fluoride may differ from iodine, folate and other substances in terms of its pharmacological effect, it shares the feature of being one of a number of successful population preventive public health strategies.

#### Bamboozling with science

Anti-fluoridation literature attempts to overwhelm readers with claims about scientific research, with figures and statistics, and with scientific terms and buzzwords. Unpacking such a dense presentation of facts, quotes and figures is beyond most people, who have neither the time nor capacity to access most of the publications required to check on the plethora of claims. A classic example of bamboozling with science is the 8-page Lifesavers Guide to Fluoridation, produced by Yiamouyiannis [102], which contains 250 references from a variety of journals, court cases, books, newsletters, symposia and newspapers, as well as several personal communications. Many of these references were subsequently used in the book 'Fluoride: the aging factor' [103], a major reference source for water fluoridation opponents. A two-year search for the cited literature by a team of people revealed less than half of the cited references to be in peer reviewed journals [54]. Almost all references were found to be incompletely cited and Yiamouyiannis was found to make superficial observations, leap to unwarranted conclusions and present a pervasive bias in his evaluation of data. However, more than two decades later the same studies continue to be cited in anti-fluoridation literature.

Opponents of water fluoridation may also attempt to bamboozle the public with language, often verging on nonsensical, yet purveying a sense of drama and foreboding. An example is shown in this quote taken from a prominent Australian fluoridation opponent:

"A maze is a model of fluoridation dental thinking, its paths (claims) leading to nowhere but neonlighted with imaginary posters of great rewards at the end of the rainbow trail which never ends, and importantly has no qualified scientific exit...The fluoridation maze hides the secrets of so-called dental science where it can be worshipped unseen with its faceless hierarchy of long on words but short of substance." [104].

Such bombastic language, combined with a litany of unsubstantiated claims, is designed to overwhelm the reader who may well find it easer to simply believe what they are being told than to try and trace the facts for themselves.

#### Moving the goalposts

Ultimately, whatever research is released showing that water fluoridation is not associated with aversive outcomes will be judged as unacceptable by fluoridation opponents. The goalposts have now been moved to such an extent that satisfying calls for supporting studies is practically impossible. Chairman of the Anti-fluoridation Association of Victoria, Glen Walker, expresses this sentiment with his statement that "there is no evidence of a scientific study proving fluoridation is perfectly safe for humans in all public circumstances" [105].

#### Paranoia, conspiracy theories and extremism

Although many opponents of water fluoridation distance themselves from extremist views, any Internet search will reveal numerous instances of these still being propounded. There is an audience for such views. Extremist arguments find fertile ground among disenfranchised, psychologically disturbed, and alienated individuals [23]. A subculture has now developed around and for such people who are believed to find psychological gratification in imagining themselves heroically in the possession of such secret and 'subversive' information [106].

Conspiracy theories relating to water fluoridation are common. Some claim that the basis of the science for water fluoridation is rooted in protecting the U.S. atomic bomb program from litigation [107]. Others argue that adding fluoride to water was pioneered by a German chemical giant to "reduce an individual's power to resist domination, by slowly poisoning and narcotizing a certain area of the brain, thus making him submissive to the will of those who wish to govern him" [108]. Still others claim that water fluoridation is part of a plot by the New World Order, a group of Illuminati, intent on taking over the world [109]. An example of paranoia is demonstrated by the following excerpt from a Christian organisation based on the preachings of William Branham:

"Fluoride is a hypnotic drug that accumulates in the body, producing schizophrenia. It was used in Russian prison camps and is harmful to dental health. The real purpose behind water fluoridation is to reduce the resistance of the masses to domination and control, and loss of liberty." [110]

The belief that water fluoridation was a communist plot to alter society was famously parodied in the movie Dr Strangelove. However, such claims were common in the 1950s at the height of the cold war between the USSR and USA. Indeed, Newbrun has described a chronology of antifluoridation propaganda in the US since the 1950s with the main themes found to reflect the social and political environment in the US at the different points in time [22].

Even some of the more prominent water fluoridation opponents often engage in what Newbrun calls the conspiracy gambit [22]. Researchers and bureaucrats are believed to be in the pay of and therefore beholden to the sugar and aluminium industries. In a pamphlet distributed by local council to residents of the rural Australian town of Deniliquin prior to a plebiscite on water fluoridation in 2004, it was claimed that: "Behind the dental and medical associations, who promote fluoridation with religious fervour, are powerful corporate and political interests" [35]. The sugary food industry, the phosphate fertiliser industry, the aluminium industry, and some governments are listed as the ominous forces behind the proponents of water fluoridation [19]. Others see water fluoridation as a cover used by generations of decision makers for the alleged failure to provide dental care to poor people. Again, no evidence is or can be offered for any of these claims.

## Summary

The list of techniques and methods described and analysed above are by no means the full extent of techniques used by water fluoridation opponents, although they are perhaps most pertinent to their promulgated literature. Common additional ploys involve neutralising politicians by massive letter writing campaigns to give the illusion of controversy, requesting public plebiscites which often have low turnouts and are dominated by people opposing change, the use of so-called experts to lend credence to anti-fluoridation claims, urging that fluoridation be delayed until better research is conducted or until the fabricated doubts can be resolved, inventing organisations with official sounding names in order to create credibility, and using public debates which give the illusion of scientific controversy and move dialogue away from scientific discussion by allowing rhetorical practices [16,111,112].

The evidence for the effectiveness of water fluoridation is incontrovertible. More than a dozen large-scale literature reviews have found water fluoridation, even against a backdrop of high discretionary fluoride use, to confer a caries preventive benefit in children. Further to this, water fluoridation and its effect on the tooth structure provides a benefit to adults across their lifespan. The situation whereby a small group of determined individuals can manage to deny half a century of science pays testimony to the power of emotional arguments and the potential of misleading propaganda. It is here that scientists must continue their stand, reinforcing the arguments for water fluoridation while using their knowledge of the literature and their understanding of anti-fluoridation tactics to assist health departments. Table 2 presents both a number of the arguments put forward by water fluoridation opponents and a possible response by water fluoridation proponents. While it is not the intention here to provide a response to every possible argument, Table 2 provides brief useful responses for the most common claims and arguments made by water fluoridation opponents. Other lists, such as those put out by the Department of Human Services Victoria, offer more complete and extensive coverage [113].

**Table 2:** Suggested responses to antifluoridationist arguments

Anti-fluoride argument	Suggested response
Water fluoridation confers no oral health benefit	Numerous systematic literature reviews from a number of countries have found water fluoridation to provide a significant caries preventive effect.
Water fluoridation causes hip fractures, cancers, Alzheimer's, reduced intelligence in children, etc.	Research finding associations between water fluoridation and various diseases offer no proof, as causality cannot be established in these studies. Water fluoridation opponents handpick studies and may misrepresent the results so as to support their views. Large-scale systematic reviews have not confirmed any associations between water fluoridation and the large list of diseases linked to it by opponents of water fluoridation.
Fluoride is a toxic poison.	Fluorine is a naturally occurring element that, like many other natural substances, can be toxic if consumed in excess. Water fluoridation ensures ingestion of fluoride well below any toxic level, both for adults and children.
Fluoride is used in rat poison and other dangerous substances.	It is dose that determines the level of toxicity. Many essential and commonly occurring elements form poisonous or toxic substances.
Numerous other countries have rejected water fluoridation.	Some other countries have elected not to introduce water fluoridation because they prefer, or already have, other approaches to improving dental health. Nonetheless, many countries do have water fluoridation and benefits are conferred to all people, including those at high risk who may not effectively use individual fluoride exposures.
Water fluoridation is supported only by 'shoddy' science.	Decades of research and hundreds of scientific articles published in peer-reviewed journals support water fluoridation. This research is so convincing that almost all major dental and health authorities support it.
There should be a public plebiscite. It is undemocratic to have water fluoridation forced upon us.	In almost all democratic systems representatives of a population are elected to make decisions on behalf of the population. Plebiscites or public referendums are not required to pass legislation that is compatible with the constitution or charter under which the country operates. Water fluoridation fits within a government's duty of care to the country's citizens.
Tooth decay has declined in countries with and those without water fluoridation. Water fluoridation makes no difference.	Declines in tooth decay have occurred as a result of changing exposures to fluoride and dietary changes. Regardless, water fluoridation reduces tooth decay above and beyond these other effects. Ecological comparisons of some countries with others offer no support for or against water fluoridation as many other factors may account for differences in disease experience from one country to the next. Water fluoridation does make a difference.
Most people do not want water fluoridation.	Independent research in most places where water fluoridation is being considered shows that people support water fluoridation. Generally, the more knowledge people have the more likely they are to support it.
Water fluoridation is costly and not economically viable.	Research has previously found water fluoridation to be cost-effective. Newer technologies have made water fluoridation cost-effective for increasingly smaller populations. In addition to being cost-effective, it is also necessary to keep in mind the reduction in dental disease and therefore the pain and suffering reduced as a result of water fluoridation.
Water fluoridation infringes freedom of choice and individual rights and is unconstitutional.	Adding fluoride to water is just one of many instances where a chemical or nutrient is added to a food or beverage for public health benefits. It already occurs in water with the addition of chlorine, which aids greatly in eliminating water borne disease, as well as in several foodstuffs. Water fluoridation sets no precedent.
Water fluoridation is being pushed on us as a result of 'big business' interests.	The scientists researching the effectiveness of water fluoridation as well as health officials and dentists do not receive money from sugar, aluminium or any other companies for their research or opinions.
There is more caries in fluoridated X than in non-fluoridated Y. This proves water fluoridation does not work.	Ecological comparisons involving the arbitrary selection of fluoridated and non-fluoridated communities or areas do not provide credible evidence of the effectiveness or otherwise of water fluoridation as any differences may be the result of other factors which are linked to tooth decay but differ across the areas. Scientific research has found water fluoridation to be effective.
We should wait until water fluoridation is proved to be safe.	Water fluoridation has been implemented in some places for more than half a century – long enough that any dangers would be apparent if they existed. The weight of evidence strongly indicates that water fluoridation is safe.

Despite more than half a century of implementation, the addition of fluoride to the water for the prevention of dental decay is still considered a controversial and debated public health measure by some segments of the population. In and of itself this state of affairs is nothing new. Campaigns have been waged over the addition of chlorine to water supplies, compulsory child immunisations, the compulsory wearing of seatbelts in cars and many other public health initiatives. However, water fluoridation suffers the inglorious distinction of being one of only a few public health initiatives to still be regularly thwarted as a result of public action based on emotional and often misleading appeals.

If Australia's National Oral Health Plan is to be implemented and water fluoridation extended to all those non-fluoridated Australian communities with populations in excess of 1000 people, a considerable effort of political will is going to be required. With decision making in most States devolved to local councils, extension of water fluoridation will occur only in a piece-meal fashion typified by a succession of emotionally charged battles between public health official and organised anti-fluoridation groups both attempting to engage the support of the public. It is therefore hoped that by better understanding the techniques and tactics of water fluoridation opponents, public health advocates, government officials, and ultimately the public, will come to dismiss many of the ant-fluoridation arguments as little more than fallacious non-science, paving the way for the extension of water fluoridation to those areas yet to benefit from its implementation.

## Competing interests

The author(s) declare that they have no competing interests.
